# Supplementary material for: Reconciling Mining with the Conservation of Cave Biodiversity: A Quantitative Baseline to Help Establish Conservation Priorities
Source: PLoS One. 2016 Dec 20;11(12):e0168348. doi: 10.1371/journal.pone.0168348 (PMC5173368; doi:10.1371/journal.pone.0168348)
Supplement: S1 Dataset — (ZIP) [file pone.0168348.s002.zip › Taxa/Serra Norte/SN_2013/N4E/Lista N4E-81.pdf]

CAVIDADE N4E-0081

Classe	Ordem	Fam/Outros	Gên/Outros	Espécie	S-U	U-S	Zon.
Annelida	Oligochaeta			jovem	2	1	E/P
Arachnida	Acari	(Oribatida)		sp.7		X	E
Arachnida	Acari	Anystidae	*Erythracarus*	sp.1		X	E
Arachnida	Acari	Eupodidae		sp.2		X	P
Arachnida	Acari	Eviphididae		sp.1		X	P
Arachnida	Acari	Ixodidae		jovem	X		E
Arachnida	Acari	Veigaiidae		sp.1		X	E
Arachnida	Amblypygi	Phrynidae	*Heterophrynus*	*longicornis*		1	E
Arachnida	Araneae	Araneidae		jovem		X	E
Arachnida	Araneae	Barychelidae		jovem	2	1	E
Arachnida	Araneae	Corinnidae		jovem	1		P
Arachnida	Araneae	Drymusidae		jovem	X		E
Arachnida	Araneae	Gnaphosidae		jovem	X		E
Arachnida	Araneae	Mimetidae		jovem	X		P
Arachnida	Araneae	Mysmenidae	*Microdipoena*	sp.1		X	E/P
Arachnida	Araneae	Pholcidae	*Leptopholcus*	sp.1	X		E
Arachnida	Araneae	Pholcidae	*Mesabolivar*	*aurantiacus*	X		P
Arachnida	Araneae	Pholcidae	*Mesabolivar*	sp.1	X		E/P
Arachnida	Araneae	Pholcidae		jovem	X	X	E
Arachnida	Araneae	Salticidae		jovem	X	X	E
Arachnida	Araneae	Segestriidae		jovem	X	X	E/P
Arachnida	Araneae	Tetragnathidae	*Leucauge*	sp.1		X	E
Arachnida	Araneae	Theridiidae	*Theridion*	sp.1	X		P
Arachnida	Araneae	Theridiosomatidae	*Plato*	sp.1	X	X	E/P
Arachnida	Opiliones	Stygnidae	*Protimesius*	*gracilis*	1	1	E
Arachnida	Pseudoscorpiones	Olpiidae		sp.1	X		E
Arachnida	Schizomida	Hubbardiidae	aff. *Rowlandius*	sp.1		X	E
Chilopoda	Scolopendromorpha	Cryptopidae	*Cryptops*	jovem		2	E
Chilopoda	Scolopendromorpha	Scolopocryptopidae	*Tidops*	sp.1		1	E
Chilopoda	Scolopendromorpha	Scolopocryptopidae	*Tidops*	sp.2		1	E
Diplopoda	Polydesmida	Cyrtodesmidae		sp.1	X	X	E/P
Diplopoda	Siphonophorida	Siphonophoridae		sp.1		X	P
Entognatha	Collembola	Entomobryidae		sp.1	X	X	E/P
Entognatha	Collembola	Entomobryidae		sp.8	X		P
Entognatha	Collembola	Paronellidae		sp.1	X	X	E/P
Entognatha	Diplura	Campodeidae		sp.1	X	X	E/P
Insecta	Blattodea	Blattellidae		jovem	2		E
Insecta	Blattodea	Polyphagidae		jovem		4	P
Insecta	Coleoptera	Chrysomelidae		sp.11	X		E
Insecta	Coleoptera	Chrysomelidae		sp.12	X		P
Insecta	Coleoptera	Ptiliidae		jovem		X	E
Insecta	Coleoptera	Scydmaenidae		sp.3		X	E
Insecta	Coleoptera	Staphylinidae	Pselaphinae	sp.24		X	E
Insecta	Coleoptera	Staphylinidae	Pselaphinae	sp.34	X		E
Insecta	Coleoptera	Staphylinidae	Pselaphinae	sp.37	X		P
Insecta	Coleoptera	Staphylinidae		sp.50	X		E
Insecta	Coleoptera	Staphylinidae		sp.54	X		E
Insecta	Diptera	Cecidomyiidae	Cecidomyiinae	sp.	X	X	E
Insecta	Diptera	Ceratopogonidae		sp.		X	P

Insecta	Diptera	Culicidae	*Culex*	sp.	X		E
Insecta	Diptera	Dolichopodidae		sp.	X		E
Insecta	Diptera	Keroplatidae	Keroplatinae	sp.	X		P
Insecta	Diptera	Phoridae	Metopininae	sp.	X		P
Insecta	Diptera	Psychodidae	Phlebotominae	sp.	X	X	E
Insecta	Diptera	Tipulidae	Tipulinae	sp.	X	X	E
Insecta	Heteroptera	Nabidae	*Arachnocoris*	jovem	X		P
Insecta	Heteroptera	Reduviidae	Reduviinae	sp.	1	1	E
Insecta	Homoptera	Cixiidae		sp.3	X	X	E
Insecta	Hymenoptera	Formicidae	*Crematogaster*	sp.1		X	P
Insecta	Hymenoptera	Formicidae	*Acromyrmex*	sp.1	X		E/P
Insecta	Hymenoptera	Formicidae	*Camponotus*	sp.1	X		E
Insecta	Hymenoptera	Formicidae	*Camponotus*	sp.2	X	X	P
Insecta	Hymenoptera	Formicidae	*Crematogaster*	sp.1	X	X	E/P
Insecta	Hymenoptera	Formicidae	*Eurhopalothrix*	sp.1		X	E
Insecta	Hymenoptera	Formicidae	*Pachycondyla*	sp.2	X	X	E/P
Insecta	Hymenoptera	Formicidae	*Paratrechina*	sp.1	X		E/P
Insecta	Hymenoptera	Formicidae	*Paratrechina*	sp.2	X		E/P
Insecta	Hymenoptera	Formicidae	*Pheidole*	sp.1	X		E
Insecta	Hymenoptera	Formicidae	*Trachymyrmex*	sp.1	X		E
Insecta	Isoptera	Rhinotermitidae	*Heterotermes*	sp.		X	E
Insecta	Isoptera	Termitidae	*Nasutitermes*	sp.	X	X	E/P
Insecta	Isoptera	Termitidae	*Termes*	sp.	X		E
Insecta	Lepidoptera	Galacticidae		sp.	X		P
Insecta	Lepidoptera	Gracillariidae		sp.		X	P
Insecta	Lepidoptera	Noctuoidea		sp.5	1	1	E
Insecta	Lepidoptera	Tineoidea		sp.1	X		E
Insecta	Lepidoptera	Tineoidea		sp.2		X	P
Insecta	Lepidoptera	Tineoidea		sp.3	X		P
Insecta	Orthoptera	Gryllidae	Nemobiinae	jovem		1	E
Insecta	Orthoptera	Phalangopsidae	*Paraclodes*	jovem	6	4	E/P
Insecta	Orthoptera	Phalangopsidae	*Phalangopsis*	sp.	5	20	P
Insecta	Psocoptera			jovem	X		P
Insecta	Thysanura	Nicoletiidae		sp.1	X		P
Gastropoda	Pulmonata	Bulimulidae	*Naesiotus*	sp.	X		P
Malacostraca	Isopoda	Armadillidae		sp.1	X		E
Malacostraca	Isopoda	Philosciidae		sp.1	X	X	E/P
Malacostraca	Isopoda	Scleropactidae		sp.1		X	E
Symphyla		Scutigerellidae		sp.	X	X	E/P
Amphibia	Anura	Leptodactylidae	*Leptodactylus*	cf. *vastus*		2	E/P
Amphibia	Anura	Strabomantidae	*Pristimantis*	cf. *fenestratus*	3		P
Amphibia	Anura			sp.		1	P
Mammalia	Chiroptera	Emballonuridae	*Peropteryx*	*kappleri*	2	2	E/P
Mammalia	Chiroptera	Phyllostomidae	*Carollia*	*perspicillata*	2		E/P
